# Effect of graft sizing in valve-sparing aortic root replacement for bicuspid aortic valve: The Goldilocks ratio

**DOI:** 10.1016/j.xjtc.2024.03.025

**Published:** 2024-04-16

**Authors:** Perry S. Choi, Amit Sharir, Yoshikazu Ono, Masafumi Shibata, Alexander D. Kaiser, Yuanjia Zhu, Alison L. Marsden, Y. Joseph Woo, Michael R. Ma, Joon Bum Kim

**Affiliations:** a Department of Cardiothoracic Surgery, Stanford University, Palo Alto, Calif; b Department of Pediatrics, Division of Pediatric Cardiology, Stanford University, Palo Alto, Calif; c Department of Thoracic and Cardiovascular Surgery, Asan Medical Center, University of Ulsan College of Medicine, Seoul, South Korea

**Keywords:** bicuspid aortic valve, valve-sparing aortic root replacement, David procedure, aortic root replacement, graft size, free-edge length, aortic valve repair

## Abstract

**Objective:**

To investigate the effect of graft sizing on valve performance in valve-sparing aortic root replacement for bicuspid aortic valve.

**Methods:**

In addition to a diseased control model, 3 representative groups—free-edge length to aortic/graft diameter (FELAD) ratio <1.3, 1.5 to 1.64, and >1.7—were replicated in explanted porcine aortic roots (n = 3) using straight grafts sized respective to the native free-edge length. They were run on a validated ex vivo univentricular system under physiological parameters for 20 cycles. All groups were tested within the same aortic root to minimize inter-root differences. Outcomes included transvalvular gradient, regurgitation fraction, and orifice area. Linear mixed effects model and pairwise comparisons were employed to compare outcomes across groups.

**Results:**

The diseased control had mean transvalvular gradient 10.9 ± 6.30 mm Hg, regurgitation fraction 32.5 ± 4.91%, and orifice area 1.52 ± 0.12 cm^2^. In ex vivo analysis, all repair groups had improved regurgitation compared with control (*P* < .001). FELAD <1.3 had the greatest amount of regurgitation among the repair groups (*P* < .001) and 1.5-1.64 the least (*P* < .001). FELAD <1.3 and >1.7 exhibited greater mean gradient compared with both control and 1.5 to 1.64 (*P* < .001). Among the repair groups, 1.5 to 1.64 had the largest orifice area, and >1.7 the smallest (*P* < .001).

**Conclusions:**

For a symmetric bicuspid aortic valve, performance after valve-sparing aortic root replacement shows a bimodal distribution across graft size. As the FELAD ratio departs from 1.5 to 1.64 in either direction, significant increases in transvalvular gradient are observed. FELAD <1.3 may also result in suboptimal improvement of baseline regurgitation.

## Graphical Abstract

**Figure:**
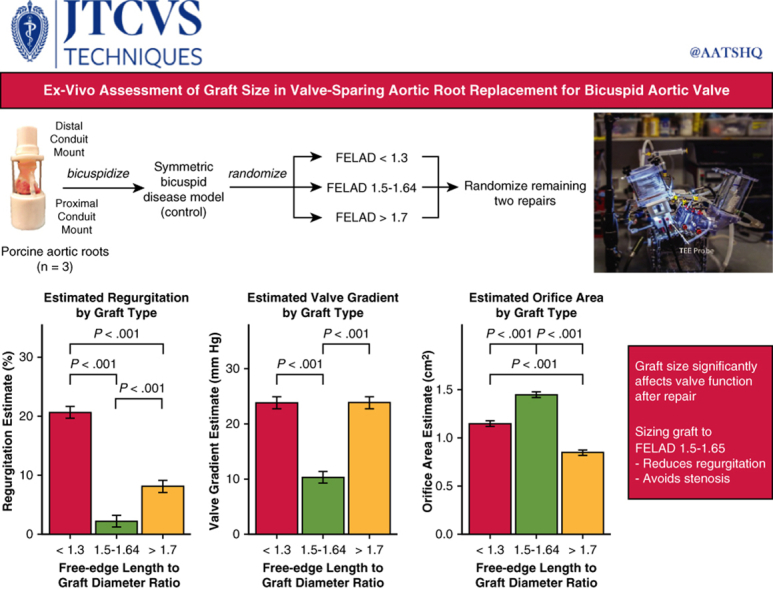

**Figure:**
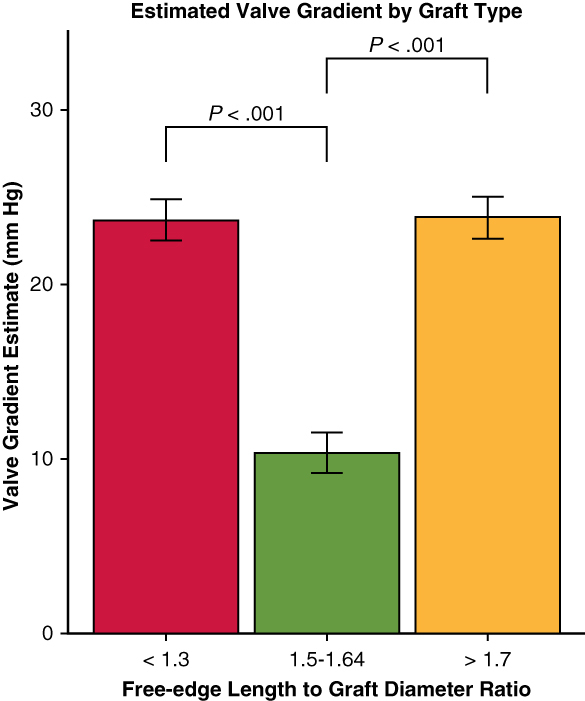
Free-edge length to graft size ratio of 1.5-1.64 results in lowest mean valve gradient.

**Central Message**

Sizing to a free-edge length to graft diameter ratio of 1.5-1.64 showed best hemodynamic performance.

**Perspective**

The merits of valve-sparing aortic root replacement in a preserved bicuspid valve are well accepted, yet the operation’s ideal parameters remain unknown. Examining the effect of graft size, the current ex vivo study reports that free-edge length-to-graft diameter ratio values beyond 1.5-1.64 in either direction lead to significant stenosis and that regurgitation may persist for values below this range.

Valve-sparing aortic root replacement (VSARR) is a well-described technique in managing aortic root pathologies when there is a relatively well-preserved native valve.^1^^,^^2^ Although more commonly reserved for patients with a tricuspid aortic valve, the technique has been expanded to patients with a bicuspid aortic valve (BAV).^3^ Recent studies looking at comparative outcomes of VSARR in patients with BAV and tricuspid aortic valves suggest similar mortality rates but potentially greater early reintervention rates in patients with BAV.^4^, ^5^, ^6^, ^7^ Aortic regurgitation is a common indication for reoperation and often occurs in the setting of cusp prolapse or root aneurysm.^6^ These studies underscore the need for tailored approaches in patients with BAV and reflect the nuanced technical challenges in managing this patient population.^8^^,^^9^ Indeed, there remains a critical knowledge gap regarding the myriad technical intricacies that surgeons must consider when contemplating whether to preserve the valve in a patient with BAV.

Previous work from our group in a simulation-based aortic valve bicuspidization model suggested that the relationship between free-edge length of a symmetric BAV and the aortic diameter directly affects valve performance.^10^ Namely, the free-edge length to aortic diameter (FELAD) ratio proved an important factor on valve performance, with evidence of stenosis for values <1.3, billowing at >1.7, and ideal conditions around 1.57. Although the computational work was designed originally for analysis in pediatric aortic valves, given the significant overlap in physical parameters, we sought in this study to assess whether a similar relationship between free-edge length and graft size would be observed in an ex vivo model for BAV VSARR.

## Methods

### Study Design

**Figure 1:**
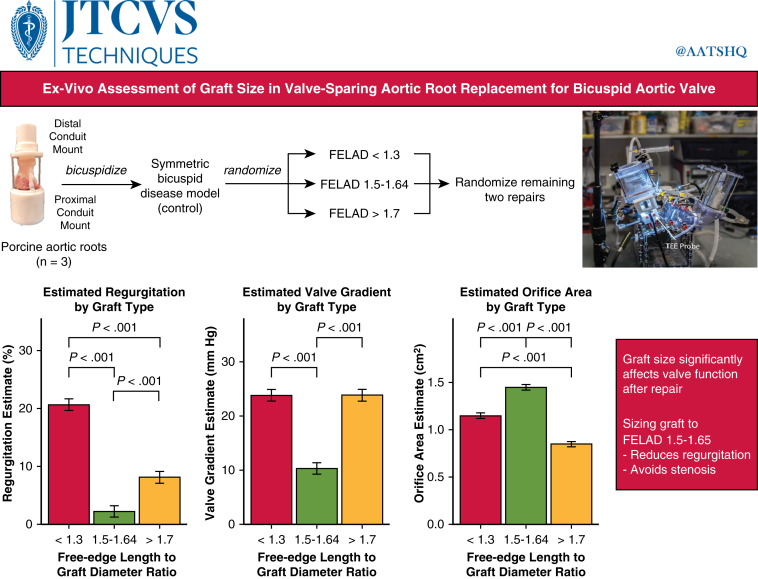
On the basis of previous simulation work, 3 porcine aortic roots were bicuspidized and tested in validated univentricular simulator for 20 runs for valve-sparing root replacement with varying FELADs: <1.3, 1.5 to 1.64, and >1.7. The control model was based on a typical diseased bicuspid valve with mild-to-moderate regurgitation. All repair conditions were tested in the same root by the same operator to minimize inter-root differences. Graft size significantly affected valve function after repair, with a free-edge length to graft ratio of 1.5 to 1.64 exhibiting ideal performance in ex vivo assessment. *FELAD*, Free-edge length to aortic/graft diameter.

On the basis of results from previous work, 3 representative groups (FELAD <1.3, 1.5-1.64, >1.7) with internal controls were replicated in explanted fetal porcine aortic roots (n = 3) using straight grafts (Figure 1).^10^^,^^11^ Groupings rather than discrete values were used because of the inability to select exact free-edge lengths from porcine specimens and availability of only even-numbered graft sizes. The trileaflet porcine aortic valves were first bicuspidized to create a diseased control model as described previously^12^ and run on a validated univentricular simulator.^13^ Specifically, the commissures of the largest cusp were moved centrally to create a 180/180 configuration, and the untouched commissure was then lowered by 10 mm to mimic a raphae. The leaflets connected to this pseudo-raphae were subsequently fused to mimic a Sievers type 1 valve with mild-to-moderate aortic regurgitation.

For the repair groups, the free-edge length of the nonfused leaflet was then measured, and 3 available graft sizes that allowed for FELAD in each of the predetermined groups were chosen. In addition to standard VSARR technique, resection of the pseudo-raphae was completed to create a fully symmetric bicuspid valve with equal geometric heights and free-edge lengths. To avoid ordering bias, the VSARR repair groups were completed in randomized fashion. All groups were tested within the same root to minimize inter-root differences. Furthermore, there were 2 operators involved in the experiment, and all repairs sewn for a given root were done by the same operator. At each stage (control, FELAD <1.3, 1.5-1.64, >1.7), these valves were run under physiological parameters (mean arterial pressure 80 mm Hg, stroke volume 90 mL, cardiac output 4.5 L/min, heart rate 75 beats/min) for 20 cycles for each condition. Measured outcomes included mean transvalvular gradient, regurgitation fraction, and orifice area.

### Statistical Analysis

To account for repeated measures within roots (each hosting multiple repair groups) and across multiple runs, linear mixed effects model was employed. Repair group (control, FELAD <1.3, FELAD 1.5-1.64, FELAD >1.7) was considered a fixed effect, and aortic roots and individual runs on the simulator were treated as random effects. The nested structure of the data, with runs being nested within roots, was explicitly accounted for in the model. To decipher differences in outcomes among the groups, post-hoc pairwise comparisons were made using the Tukey honestly significant difference test. R, version 4.3.1 (R Foundation for Statistical Computing) was used for analysis.

## Results

For the 3 porcine aortic roots used in the study, the mean aortic diameter (measured at the sinotubular junction) was 29.3 ± 2.52 mm (Table 1). The mean leaflet free-edge length was 39.3 ± 2.5 mm and the mean FELAD 1.34 ± 0.03. These roots were tested on the univentricular simulator at a mean aortic pressure of 86.6 ± 6.2 mm Hg. The diseased control model had mean regurgitation fraction 32.5 ± 6.3%, mean transvalvular gradient 10.9 ± 6.3 mm Hg, and mean orifice area 1.52 ± 0.1 cm^2^.

**Table 1:** Valve characteristics for native aortic root and bicuspidized control across aortic roots

Characteristic	Overall	Root 1∗	Root 2∗	Root 3∗	*P*†
Native aortic root					
Aortic diameter, mm	29.3 ± 2.5	30.0	26.0	32.0	<.001
Free-edge length, mm	39.3 ± 2.5	40.0	36.0	42.0	<.001
Free-edge length to aortic diameter ratio	1.34 ± 0.03	1.33	1.38	1.31	<.001
Bicuspidized control					
Valve gradient, mm Hg	10.9 ± 6.3	6.6 ± 0.8	19.7 ± 0.4	6.3 ± 0.2	<.001
Valve regurgitation fraction, %	32.5 ± 4.9	27.5 ± 3.1	35.7 ± 1.5	34.4 ± 4.7	<.001
Valve orifice area, cm^2^	1.52 ± 0.12	1.59 ± 0.04	1.36 ± 0.01	1.60 ± 0.01	<.001
Repair groups					
Graft size (FELAD <1.3)	32.7 ± 1.9	34	30	34	<.001
Graft size (FELAD 1.5-1.64)	25.3 ± 1.0	26	24	26	<.001
Graft size (FELAD >1.7)	21.3 ± 2.5	22	18	24	<.001

*FELAD*, Free-edge length to aortic/graft diameter.

∗ Mean ± standard deviation.

† Kruskal-Wallis rank sum test.

For the repair groups, mean graft size for the FELAD <1.3 group was 32.7 ± 1.9 mm, 25.3 ± 1.0 mm for FELAD 1.5-1.64, and 21.3 ± 2.5 mm for FELAD >1.7 (Table 2). Given a mean free-edge length of 39.3 ± 2.5 mm, the respective mean FELAD across groups was 1.20 ± 0.02, 1.55 ± 0.05, and 1.86 ± 0.11. Mean regurgitation fraction was 20.7 ± 7.3% for FELAD <1.3, 2.3 ± 2.1% for FELAD 1.5-1.64, and 8.2 ± 4.0% for FELAD >1.7. The mean gradient was 23.7 ± 10.5 mm Hg for FELAD <1.3, 10.3 ± 4.9 for FELAD 1.5-1.64, and 23.8 ± 8.4 for FELAD >1.7. Lastly, mean valve orifice area was 1.15 ± 0.16 cm^2^ for FELAD <1.3, 1.45 ± 0.19 cm^2^ for FELAD 1.5-1.64, and 0.85 ± 0.05 for FELAD >1.7.

**Table 2:** Valve characteristics across graft size groups

Characteristic	Control	FELAD <1.3∗	FELAD 1.5-1.64∗	FELAD >1.7∗	*P*†
Aortic/graft diameter, mm	29.3 ± 2.5	32.7 ± 1.9	25.3 ± 1.0	21.3 ± 2.5	<.001
Free-edge length, mm	39.3 ± 2.5	39.3 ± 2.5	39.3 ± 2.5	39.3 ± 2.5	>.9
Free-edge length to aortic/graft diameter ratio	1.34 ± 0.03	1.20 ± 0.02	1.55 ± 0.05	1.86 ± 0.11	<.001
Valve gradient, mm Hg	10.9 ± 6.3	23.7 ± 10.5	10.3 ± 4.9	23.8 ± 8.4	<.001
Valve regurgitation fraction, %	32.5 ± 4.9	20.7 ± 7.3	2.3 ± 2.1	8.2 ± 4.0	<.001
Valve orifice area, cm^2^	1.52 ± 0.12	1.15 ± 0.16	1.45 ± 0.19	0.85 ± 0.05	<.001

*FELAD*, Free-edge length to aortic/graft diameter.

∗ Mean ± standard deviation.

† Kruskal-Wallis rank sum test.

**Figure 2:**
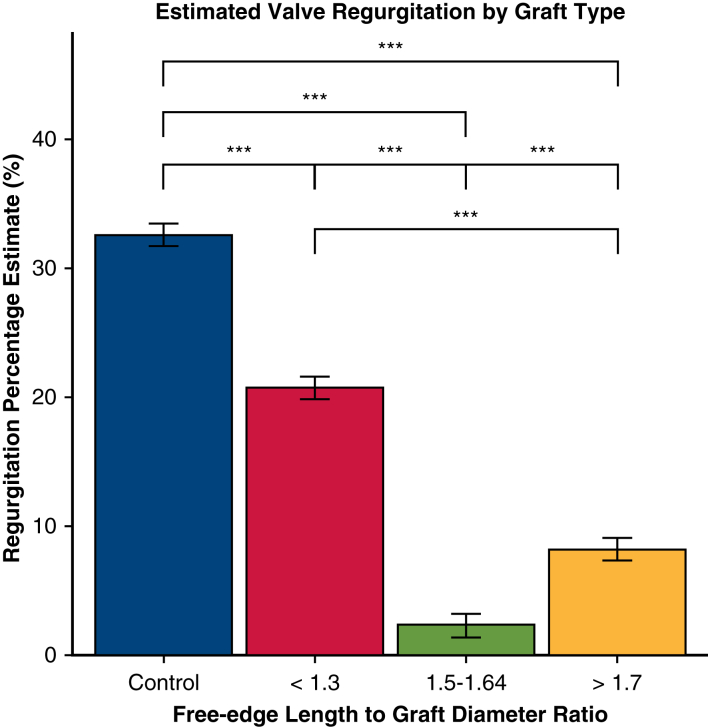
Valve regurgitation based on free-edge length to graft diameter ratio. ∗∗∗*P* < .001. All repair groups had significantly improved regurgitation compared with control. Pairwise comparison showed that the <1.3 group had significantly greater regurgitation than both the 1.5 to 1.64 and >1.7 groups. The 1.5 to 1.64 group had significantly lower regurgitation than the >1.7 group. Error bars denote standard error from linear mixed effects model.

Linear mixed effects modeling revealed all repair groups had significantly improved regurgitation compared with control (estimate −11.9% for FELAD <1.3, −30.3% for FELAD 1.5-1.64, −24.3% for FELAD >1.7, *P* < .001) (Figure 2). Pairwise comparison showed that FELAD <1.3 had significantly greater regurgitation than both FELAD 1.5-1.64 (estimate 18.4%, *P* < .001) and FELAD >1.7 groups (estimate 12.5%, *P* < .001). Moreover, FELAD 1.5-1.64 had significantly lower regurgitation than the >1.7 group (estimate −5.9%, *P* < .001).

**Figure 3:**
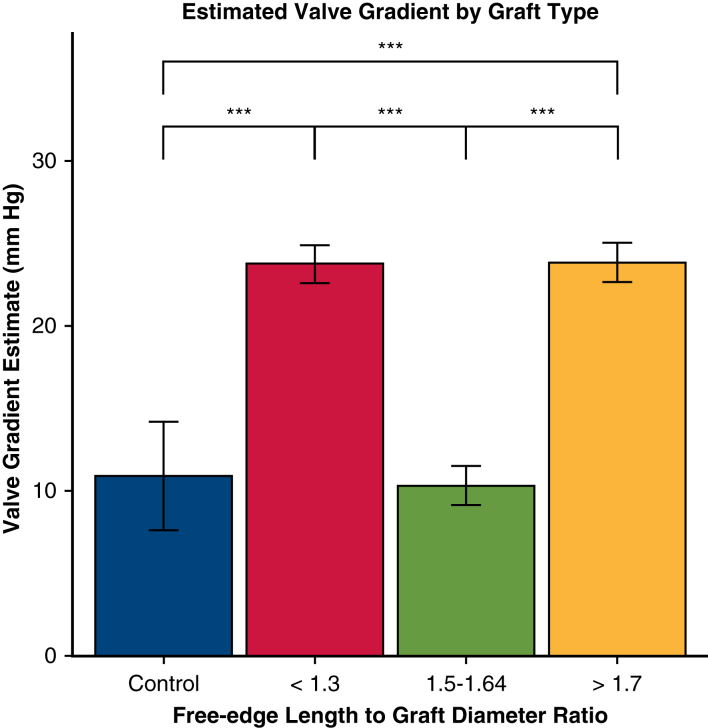
Transvalvular gradient based on free-edge length to graft diameter ratio. ∗∗∗*P* < .001. Linear mixed effects modeling across repair groups showed significantly higher mean transvalvular gradient for the <1.3 and >1.7 groups compared with control, whereas the 1.5 to 1.64 group was not significantly different from control (*P* = .64). Pairwise comparisons confirmed these results and showed that the 1.5 to 1.64 group had significantly lower mean transvalvular gradient than both the <1.3 and >1.7 groups. Error bars denote standard error from linear mixed effects model.

The mean transvalvular gradient was significantly elevated compared with control for both FELAD <1.3 (estimate 12.9 mm Hg, *P* < .001) and FELAD >1.7 (estimate 13.0 mm Hg, *P* < .001) (Figure 3). However, the mean gradient for the FELAD1.5-1.64 group was not significantly different from control (*P* > .05). Pairwise comparisons confirmed these results and showed that FELAD 1.5-1.64 had significantly lower transvalvular gradient than both the <1.3 (estimate −13.4 mm Hg, *P* < .001) and >1.7 groups (estimate −13.5 mm Hg, *P* < .001). The mean gradient was not significantly difference between the FELAD <1.3 and FELAD >1.7 groups (*P* > .05, Figure 2).

**Figure 4:**
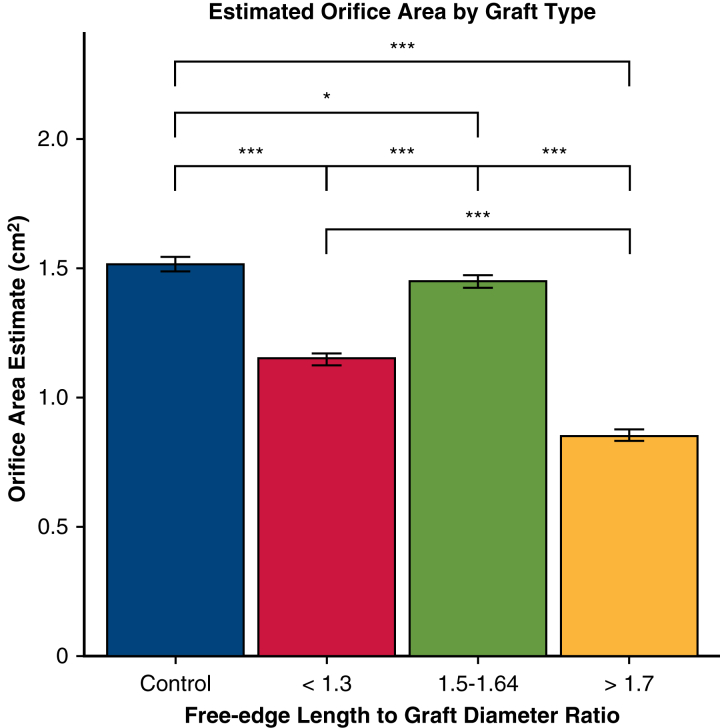
Valve orifice area based on free-edge length to graft diameter ratio. ∗*P* < .05, ∗∗∗*P* < .001. All repair groups had significantly smaller orifice area compared with the control group. Pairwise comparison showed that the 1.5 to 1.64 group had significantly larger orifice area than the <1.3 and >1.7 groups, whereas the >1.7 group had significantly smaller orifice area than the <1.3 group. Error bars denote standard error from linear mixed effects model.

Notably, all repair groups had significantly smaller orifice area compared with control (estimate −0.37 cm^2^ for FELAD <1.3, −0.07 cm^2^ for FELAD 1.5-1.64, −0.66 cm^2^ for FELAD >1.7, *P* < .01) (Figure 4). Pairwise comparison showed that FELAD 1.5-1.64 group had significantly larger orifice area than the FELAD <1.3 (estimate 0.30 cm^2^, *P* < .001) and FELAD >1.7 groups (estimate 0.60 cm^2^, *P* < .001), and the FELAD >1.7 group had significantly smaller orifice area than the <1.3 group (*P* < .001).

## Discussion

The present ex vivo study confirms our previously published simulated results in the context of VSARR for BAV. As expected, there was a clear relationship between FELAD ratio and valve performance, with the FELAD 1.5 to 1.64 group demonstrating significantly lower transvalvular gradient and greater orifice area compared with the FELAD <1.3 group. Moreover, the FELAD >1.7 group had significantly more regurgitation compared with the 1.5 to 1.64 group, which aligns well with the increased billowing previously observed in simulated results.

Clinically, these results are intuitive. The larger the graft one uses (lowering the FELAD for a given leaflet configuration), the more the leaflets are stretched and the less surface area available for coaptation. In a trileaflet semilunar valve, such stretching eventually results in central noncoaptation causing regurgitation. In a bileaflet semilunar valve, stretching results in progressive stenosis as the free-edge leaflet becomes too short relative to the annular circumference to open properly. Hence, one may posit that increased mean gradient is associated with larger graft sizes (ie, lower FELAD).

Interestingly, however, the FELAD >1.7 group also exhibited significantly greater transvalvular gradient and lower orifice area compared with the control and FELAD 1.5 to 1.64 groups. These unexpected results are most likely the result of the presence of excess leaflet tissue, as significant crowding of material inside the annulus (as opposed to in the sinuses) in systole was observed in the FELAD >1.7 group. This is consistent with the observation that the FELAD >1.7 group had the smallest orifice area. These findings point instead to a bimodal distribution of performance, suggesting that both oversizing and undersizing the graft may lead to significant stenosis.

Another unexpected finding was increased valve regurgitation for the FELAD <1.3 group when compared with the FELAD 1.5 to 1.64 group. Whereas increased stenosis is expected at lower FELAD ratios as the result of decreased excursion of the leaflets, regurgitation is not. Indeed, one can imagine that decreased leaflet excursion during the cardiac cycle may lead to decreased regurgitation, if anything. Given that regurgitation for the FELAD <1.3 group was still significantly lower than that for the control group, this finding may simply be attributable to fact that the diseased control model had baseline moderate regurgitation.

In other words, even though there is decreased leaflet excursion compared with the FELAD 1.5 to 1.64 group, the diseased control model’s baseline deficiency in coaptation may have been only partially addressed in the FELAD <1.3 group. Furthermore, one can imagine that the tighter the leaflets are pulled in a bicuspid valve, there is not only reduced leaflet excursion but also decreased coaptation surface available as the leaflet progressively gets pulled radially. Importantly, the use of a diseased control model was deliberately chosen a priori to best mimic the conditions an operator would face when conducting a VSARR for BAV, thereby improving relevance of the experiment’s results, as well as establishing an internal control to improve validity of comparison across groups. However, the control model still has important limitations, such as nonincorporation of leaflet and annular dilation typically observed in patients undergoing VSARR. It will be important in follow-up studies to modify computational simulation with a diseased control model to clarify and confirm the current study results.

An important study limitation is incomplete sampling across the spectrum of FELAD. Although previous work suggests division into 3 subgroups would be sufficient as the result of a predicted bimodal distribution, the precision of results would be enhanced with additional subgroups. Namely, a bimodal distribution for stenosis was not expected, so future studies are planned to assess performance of valves in the gaps of FELAD values on either side of the 1.5 to 1.64 group. This will help further delineate the inflection points across these outcomes to better inform operators facing this complex pathology.

## Conclusions

Graft size significantly affects valve performance after VSARR in the context of BAV with mild-to-moderate regurgitation. Sizing to a FELAD ratio of 1.5 to 1.64 can significantly reduce regurgitation without increasing stenosis. However, sizing to a FELAD <1.3 or >1.7 may significantly increase transvalvular gradient and decrease valve orifice area. Lastly, sizing to FELAD <1.3 may reduce valve regurgitation to a lesser extent than both FELAD 1.5 to 1.64 and >1.7.

## Conflict of Interest Statement

The authors reported no conflicts of interest.

The *Journal* policy requires editors and reviewers to disclose conflicts of interest and to decline handling or reviewing manuscripts for which they may have a conflict of interest. The editors and reviewers of this article have no conflicts of interest.
